# Topological Valley Transport in Two-dimensional Honeycomb Photonic Crystals

**DOI:** 10.1038/s41598-018-20001-3

**Published:** 2018-01-25

**Authors:** Yuting Yang, Hua Jiang, Zhi Hong Hang

**Affiliations:** College of Physics, Optoelectronics and Energy & Collaborative Innovation Center of Suzhou Nano Science and Technology, Soochow University, Suzhou, 215006 China

## Abstract

Two-dimensional photonic crystals, in analogy to AB/BA stacking bilayer graphene in electronic system, are studied. Inequivalent valleys in the momentum space for photons can be manipulated by simply engineering diameters of cylinders in a honeycomb lattice. The inequivalent valleys in photonic crystal are selectively excited by a designed optical chiral source and bulk valley polarizations are visualized. Unidirectional valley interface states are proved to exist on a domain wall connecting two photonic crystals with different valley Chern numbers. With the similar optical vortex index, interface states can couple with bulk valley polarizations and thus valley filter and valley coupler can be designed. Our simple dielectric PC scheme can help to exploit the valley degree of freedom for future optical devices.

## Introduction

In the electronic crystal system, a pair of inequivalent energy extrema exhibits in the momentum space, called valleys^1,2^. Valleytronics aims to use the valley index of carriers, a degree of freedom that is analogous to spin in spintronics, to process information in modern electronic devices^3,4^. Many interesting phenomena of valleytronics were theoretically predicted and experimentally observed^5–20^. For example, to modulate the different valleys by uniaxial stress in aluminum arsenide^8^ or rotating magnetic field in bismuth^9^, valley polarization can be generated. Valley-selective excitation has been demonstrated in single layer of graphene and transition metal dichalcogenides^10–12^. Recently, theorists have proposed that topological gapped bilayer graphene may possess protected edge states or interface states^13–16^, where valley polarized current is conducted and backscattering is suppressed. There are strong experimental evidences indicating the existence of topologically protected edge states in bilayer graphene structures with AB-BA stacked domain walls^17–20^. However, because of the inevitable atomic-scale imperfections and the difficulty of distinguishing valley degree of freedom in electronic system, direct observation of valley transport is still challenging, which prevents the future applications of valley polarization and topological edge states.

In analogy to electronic wave, classical sound or light wave are also considered to be a perfect platform to reveal the valley polarization and topological nature of materials^21^. Different from the methods for selectively stimulating valleys in electronic structure^22,23^, in photonic graphene (where coupled optical fibers were organized in a honeycomb lattice), pseudospin-mediated vortices of valleys were generated and experimentally observed by two-beam excitations^24^. Due to the flexible design of domain walls, the valley-polarized selection has been observed experimentally in sonic crystals^25^, where the topological valley transport of sound were produced. Valley-Hall photonic topological insulators were also numerically demonstrated using two-dimensional (2D) photonic crystal (PC) structures^26^. A domain wall between such two photonic topological insulators can support edge states nearly without backscattering^27–31^.

In this manuscript, we study the topological nature of 2D PCs with honeycomb lattice. A honeycomb PC with different atoms in the unit cell bears similarity to gapped bilayer graphene with AB/BA stacking, where inversion symmetry is also broken. Thus, such a simple PC structure is a perfect platform to examine valley topological features in bilayer graphene. Using a designed chiral stimulus with different optical vortex index^32^, the excitation of inequivalent bulk valley states are demonstrated. We verify the valley polarization from the chiral phase distributions and the emitting beams out of the PCs. Topological interface states as in AB/BA bilayer graphene domain walls are realized and their backscattering immune propagation is visualized. Since the mode of topological interface states and bulk valley states are both of optical vortices, such topological interface states can be utilized to select bulk valleys. As a result, the valleytronic devices, including a valley filter and a valley coupler are numerically demonstrated by coupling between interfacial domain walls and bulk PC valley materials. Our PC platform not only demonstrates valley physics predicted from bilayer graphene, but also provides a scheme to manipulate valley degree of freedom in PCs. It will help the design of future optical devices.

## Results

### Chiral valley state

**Figure 1:**
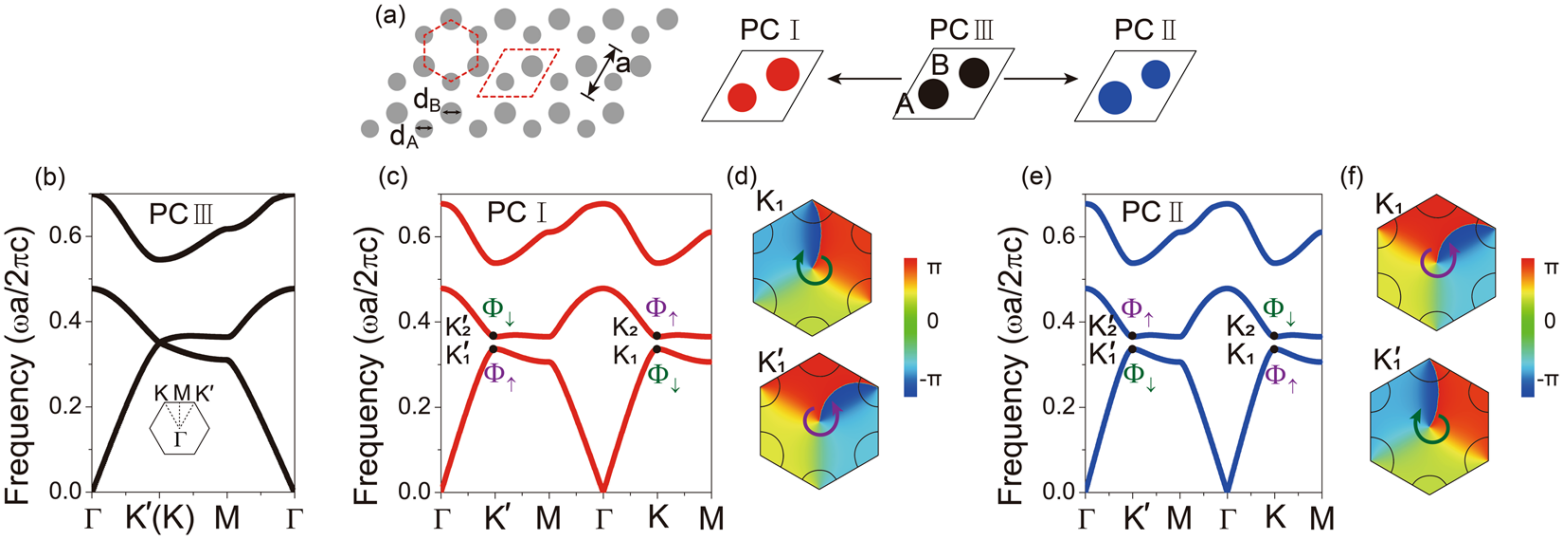
(**a**) Schematic of 2D valley PC system with dielectric cylinders embedded in air background. (**b**) The band dispersion with the Dirac cones at *K* and *K*′ points for PC III which has the same cylinders in one unit cell. (**c**,**e**) As the diameters of cylinders in a unit cell are not identical in both PC I and II, photonic band gap appears by breaking *C*_*6v*_ symmetry. (**d**,**f**) The phase distributions reveal that *K*_1_ and ${K^{\prime} }_{1}$ have opposite chirality. The *K*_1_ and ${K^{\prime} }_{1}$ states have inverted eigenfield phase variations as denoted by pseudospin down Φ_↓_ and up Φ_↑_ for PC I, while pseudospin up Φ_↑_ and down Φ_↓_ for PC II.

Figure 1(a) plots the schematic of the 2D honeycomb PC consisting of dielectric cylinders (in gray) embedded in air background. Its unit cell as marked by red dashed rhombus comprises of two dielectric cylinders (A and B) with infinite height, whose relative permittivity $\varepsilon =7.5$. The polarization under consideration is transverse-magnetic (TM), with the electric field parallel to the cylinder axis. We consider the diameters of the two cylinders in a unit cell independently. When they are of the same value, i.e. ${d}_{A}={d}_{B}=0.367a$ (*a* is the lattice constant), the corresponding photonic band structure is calculated numerically using finite-element methods depicted in Fig. 1(b). Degenerated valleys appear around the corners of the first Brillouin zone (*K* and *K*′ points). This case is denoted as PC III, which is similar to monolayer graphene by replacing each carbon atom with an infinitely long dielectric cylinder. By breaking *C*_*6v*_ symmetry of the lattice, the degeneracy of valleys is lifted and a complete photonic band gap appears (see Fig. 1(c) and (e)). There are various ways to break *C*_*6v*_ symmetry of a honeycomb lattice^33^ and hereby the inversion symmetry is broken by considering different diameters of the two cylinders. For instance, we consider the structure referring as PC I by decreasing the diameter of cylinder A while increasing the diameter of cylinder B. Materials and other properties remain unchanged between PC I and III. The corresponding photonic band structure for PC I with *d*_*A*_ = 0.333*a*, *d*_*B*_ = 0.4*a* is plotted in Fig. 1(c). It displays two pairs of valley states *K* and *K*′ at the normalized frequency 0.337 and 0.365*c*/*a* (*c* is the speed of light in the vacuum), where the lower and higher frequency state at *K* (*K*′) point is referred as *K*_1_ (${K^{\prime} }_{1}$) and *K*_2_ (${K^{\prime} }_{2}$) respectively. The valley states at *K* and *K*′ points are time-reversal symmetry protected but inequivalent. The phase distributions of the eigenfields of *K*_1_ and ${K^{\prime} }_{1}$ are shown in Fig. 1(d). By only breaking inversion symmetry, *C*_3*v*_ symmetry is still maintained. At the rotation center of *C*_3*v*_, the phase variation of the *K*_1_ valley decreases clockwise as illustrated by a green arrow. We define this photonic state as pseudospin down Φ_↓_. In contrast to *K*_1_, the phase variation of the ${K^{\prime} }_{1}$ valley rotates anticlockwise as illustrated by a purple arrow (denoted as pseudospin up state Φ_↑_). The chirality of *K*_1_ and ${K^{\prime} }_{1}$ is reversed, where similar properties can be found in electronic valley states. The phase distributions of *K*_2_ and ${K^{\prime} }_{2}$ valleys can also be numerically obtained, where *K*_2_ is of pseudospin up state Φ_↑_ and ${K^{\prime} }_{2}$ is of pseudospin down state Φ_↓_.

If the changes of diameters for cylinders A and B are inverted, we can do similar analysis. PC II is realized by setting ${d}_{A}=0.4a$ and ${d}_{B}=0.333a$, which can basically be regarded as swapping the positions of cylinders A and B in PC I. Actually, PC I and II are identical to each other if one is rotated by 180° around the unit cell center. Correspondingly, they have identical photonic band structures, while the eigenmode profiles of phases are opposite. As shown in Fig. 1(e) and (f). The valley state *K*_1_ (${K^{\prime} }_{1}$) of PC II is pseudospin up Φ_↑_ (down Φ_↓_) state. A similar structure was proposed by using valley dielectric PCs to engineer the spin-orbital interaction of light in nanophotonics^29^.

### Valley polarization

**Figure 2:**
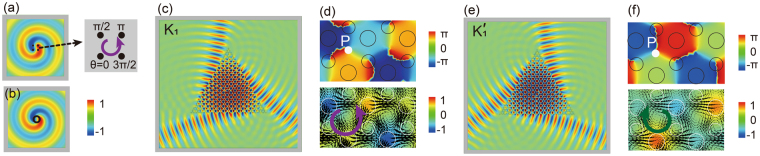
(**a**) The chiral source is constructed by a four-antenna array with discontinuous phases, where the phase decreases anticlockwise between neighbors by *π*/4. The simulated field distribution is identical with the optical mode with vortex index *m* = 1 as shown in (**b**). Valley states for *K*_*1*_ and ${K^{\prime} }_{1}$ are selectively excited by chiral sources with optical vortex index *m* = 1 (**c**) and *m* = −1 (**e**), respectively. The distributions of phases reveal opposite singular points at the rotational center of a unit cell in the top panels of (**d**) and (**f**). Poynting vectors clearly display the chiral properties of valley states in the bottom panels.

The valley indices can be regarded as degree of freedom for valleytronic devices because of the spatial separation in momentum space. In order to use the valley degree to process information, an important step is to identify the inequivalent valleys. In electronic system, circularly polarized light couples only to a specific valley and thus different valley can be excited^34^. If a special electromagnetic stimulus can be designed, which only couples to one specific valley index, bulk properties of valley PCs can be easily explored. To generate the valley-selective optical excitation, we design a chiral source using a four-antenna array^35^ which is easily constructed in both simulations and experiments. The initial phases of the electromagnetic wave in neighboring antennas decrease by *π*/4 anticlockwise, whose field pattern (shown in Fig. 2(a)) is identical to that of optical mode with optical vortex index *m* = 1 (shown in Fig. 2(b)). The *m* = 1 mode can be considered to carry the *j* = 1 orbital angular momentum. As depicted in Fig. 2(c), a source with *m* = 1 locates at the center of a regular triangle structure made of PC II as discussed in Fig. 1. The Φ_↑_ valley state at *K*_1_ point couples exclusively to a chiral optical source with *m* = 1 and is well excited at the normalized frequency 0.335*c*/*a*. In the contrast, the ${K^{\prime} }_{1}$ state, even though at the same frequency but with a reversed vortex index, is suppressed. A source with optical vortex index *m* = −1 can be constructed with a *π*/4 phase increase anticlockwise among neighboring antennas. The ${K^{\prime} }_{1}$ valley state of pseudospin down can thus be excited, as displayed in Fig. 2(e). The selective excitation of a specific valley state is visualized from simulations. The phase singular points are recognized in the zoom-in phase distribution in the top panels of Fig. 2(d) and (f), which are the rotating centers of *C*_*3v*_ symmetry of the unit cell (see as P point). If we further calculate the Poynting vector $\overrightarrow{S}=\mathrm{Re}[\overrightarrow{E}\times {\overrightarrow{H}}^{\ast }]/2$ distributions, as shown in the bottom panels of Fig. 2(d) and (f), $\overrightarrow{S}$ rotates anticlockwise/clockwise around these phase singular points, indicating optical vortex nature of the corresponding valley state.

Inter-valley scattering is negligible at the PC boundary by using a regular triangular-shaped structure, where its surface orientation is selected along the $\Gamma M$ direction^32^. The *K*_1_ and ${K^{\prime} }_{1}$ valley states refract partly to itself and partly to the free space at the boundaries. The emission angle follows the conversion of momentum parallel to the crystal interface. We can detect the valley polarization excited by the refracted beams out of the PC structure. The refracted beams as plotted in Fig. 2(c) and (e) are obviously different. From the refractive direction of the three beams, we can also distinguish the excited state belonging to *K* or *K*′ valley.

**Figure 3:**
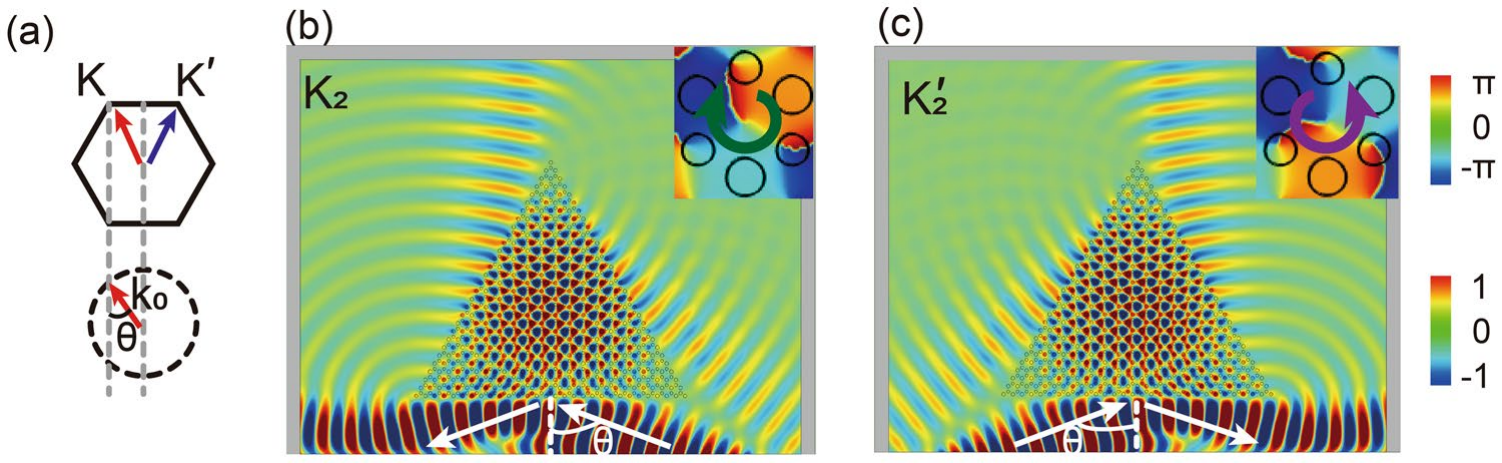
(**a**) The schematic displays the theory of the valley-selection excitation from the external beam. (**b**,**c**) The *K*_2_ and ${K^{\prime} }_{2}$ valley states are selectively excited by Gaussian beams at different incident directions respectively.

In general, valley states can be selectively excited if an electromagnetic source that matches with the eigenmode profile of that valley polarization state can be designed. If a specific bulk valley state is solely excited, the internal phase distribution as well as the refractive beam out of the bulk PC structure will be determined as well. Such properties give us methods to determine the excited valley state. Valley-dependent bulk excitation and wave transport were reported based on the distinct iso-frequency contour shapes at different frequencies^36,37^. Here we introduce a different mechanism. By increasing working frequency to *K*_2_/${K^{\prime} }_{2}$, we can also excite *K*_2_ and ${K^{\prime} }_{2}$ states by a proper choice of excited source. Since the system has time reversal, the valley states can also be excited by an incident light beam from a special angle. The schematic in Fig. 3(a) displays the physical mechanism for the incident beam excited the valley states, where the momentum is parallel to the PC boundary^25^ i.e. $k={k}_{0}\,\sin \,\theta$. Here *k*_0_ is the wavevector in air, *θ* is the incident angle of the beam and *k* is the projection of *K*/*K*′ on the boundary. As shown in Fig. 3(b), a Gaussian beam impinges on the triangular PC I structure and reflects partly at frequency 0.367*c*/*a* with incident angle $\theta ={\sin }^{-1}(k/{k}_{0})={65.2}^{\circ }$. Now the *K*_2_ valley state is excited, which can be justified from the clockwise rotating phase distribution (see the inset). When the beam is incident at another direction from the boundary, the ${K^{\prime} }_{2}$ valley state is excited. Please be noted that the rotating phase distribution of ${K^{\prime} }_{2}$ is reversed compared to that of *K*_2_ discussed previously.

There are two degrees of freedom for the bulk valley states of the honeycomb PC: the frequency (*K*_1_ or *K*_2_ state) and the optical vortex index of its mode profiles. By manipulating both degrees of freedom simultaneously, we can explore various properties related to the valley PC structure and design the valleytronic devices accordingly.

### Topological interface states

**Figure 4:**
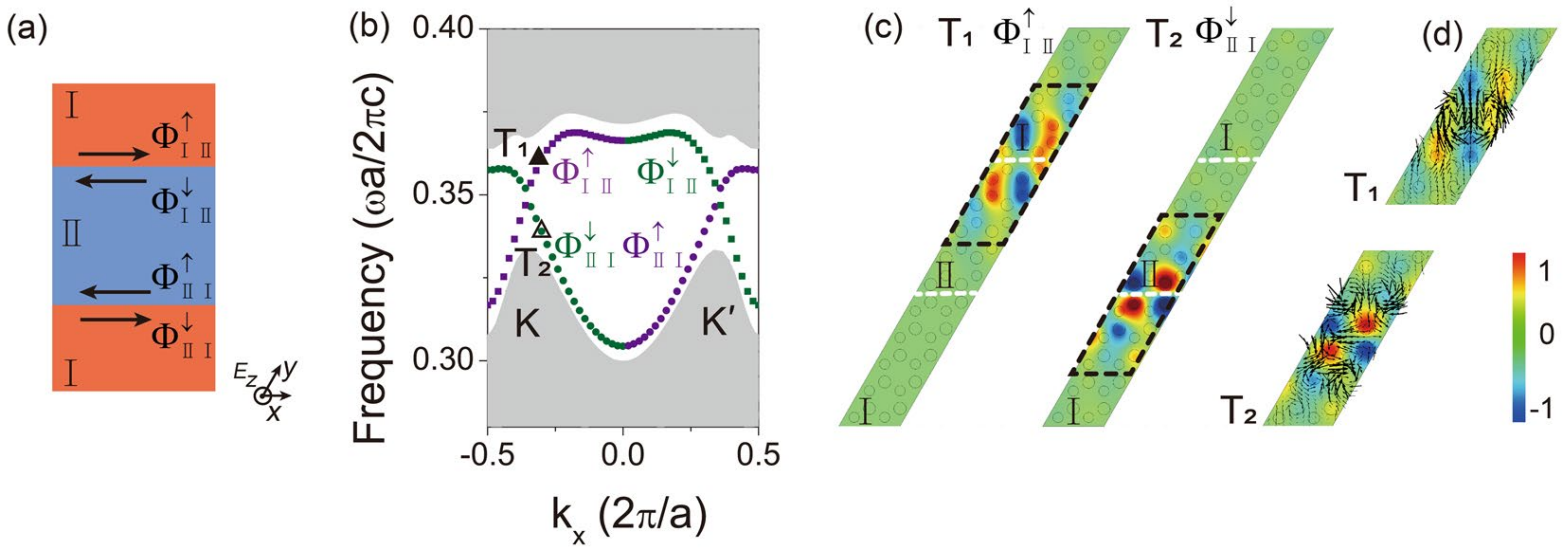
(**a**) Schematic of two pairs of counter-propagating topological valley interface states. Two interfaces for PC I and II possess pseudospin up (${{\rm{\Phi }}}_{{\rm{I}}\,{\rm{I}}\,{\rm{I}}}^{\uparrow }$, ${{\rm{\Phi }}}_{{\rm{I}}\,{\rm{I}}\,{\rm{I}}}^{\uparrow }$) and pseudospin down (${{\rm{\Phi }}}_{{\rm{I}}\,\,{\rm{II}}}^{\downarrow }$, ${{\rm{\Phi }}}_{{\rm{II}}\,{\rm{I}}}^{\downarrow }$ ) states. (**b**) The projected band structure is calculated to investigate the properties of topological edge modes. These modes are indicated by rectangular and circular dots for Φ_I II_ and Φ_II I_ respectively. (**c**) Topological states (${{\rm{\Phi }}}_{{\rm{I}}\,\,{\rm{II}}}^{\uparrow }$, ${{\rm{\Phi }}}_{{\rm{II}}\,{\rm{I}}}^{\downarrow }$) are confined well at the interfaces observed from distributions of eigenmodes at T_1_ and T_2_ points. (**d**) The spatial distributions of Poynting vectors manifest the vortex nature for T_1_ and T_2_ points.

Similar to electronic system, we can also use valley Chern numbers to define the topology of PC system. The valley Chern numbers are quantized and remain unchanged under arbitrary continuous deformation of the system. They can be calculated by integrating the Berry curvature over the entire Brillouin zone^15,38^. For honeycomb lattice, when inversion symmetry is broken (by considering different diameters of cylinders A and B in the unit cell), inequivalent *K* and *K*′ valleys as well as photonic band gaps are obtained. The Berry curvatures have opposite values in two valleys. The valley Chern numbers *C* of these two valleys for PC I, aforementioned in Fig. 1, have the same magnitude but with opposite sign: ${C}_{{\rm{I}}}^{K}=-1/2$, ${C}_{{\rm{I}}}^{K^{\prime} }=1/2$ ^26,27,30^. The bulk photonic band structure of PC II is identical to that of PC I because only an inversion symmetry operation is needed. However, the band topology of PC I and II is different. We obtain the valley Chern numbers of PC II as ${C}_{{\rm{II}}}^{K}=1/2$, ${C}_{{\rm I}{\rm I}}^{K^{\prime} }=-1/2$. In bilayer graphene systems^13–20^, researchers must manipulate the interface dislocation of AB/BA domain walls to construct a backscattering immune channel. Here, we can realize the similar effect by a much simpler structure of PC domain wall through connecting PC I along with PC II. The valley Chern numbers in these two structures are reversed, indicating the occurrence of band inversion between PC I and II. The topological phase transition can take place at the interface. Though the sum of valley Chern number differences of both PC systems is equal to zero, the valley Chern number difference Δ*C* is quantized, i.e. ${\rm{\Delta }}{C}_{{\rm{I}}\,{\rm{II}}}^{K}={C}_{{\rm{I}}}^{K}-{C}_{{\rm{II}}}^{K}=-1$, ${\rm{\Delta }}{C}_{{\rm{I}}\,{\rm{II}}}^{K^{\prime} }={C}_{{\rm{I}}}^{K^{\prime} }-{C}_{{\rm{II}}}^{K^{\prime} }=1$ across the boundary. Hence, the quantized valley Chern number difference ensures the existence of gapless interface states at the domain wall between the two inverted structures. Figure 4(a) illustrates the schematic of the domain wall, where PC I and II are indicated in red and blue respectively. The system has two pairs of counter-propagating topological interface states along the interface channels, denoted as (${{\rm{\Phi }}}_{{\rm{I}}\,{\rm{II}}}^{\uparrow }$, ${{\rm{\Phi }}}_{{\rm{I}}\,{\rm{II}}}^{\downarrow }$) and (${{\rm{\Phi }}}_{{\rm{{\rm I}}}{\rm{{\rm I}}}\,{\rm{I}}}^{\uparrow }$, ${{\rm{\Phi }}}_{{\rm{II}}\,{\rm{I}}}^{\downarrow }$) states. Φ_I II_ (Φ_II I_) denotes the situation that PC I stands above (below) PC II and the arrows indicate their chirality nature. We consider a supercell of both PC I and II with the wave vector along the *k*_*x*_ direction in simulation. This supercell consists of five unit cells for both PC I and PC II along the *y* direction and the periodic boundary conditions along *x* and *y* directions are applied. The calculated band structure is shown in Fig. 4(b). The bulk bands for both PCs are painted in gray, and the remains in the band structures are the dispersion of interface states, where the rectangular and circular dots represent Φ_I II_ and Φ_II I_ respectively. The eigenfield distributions as indicated by T_1_ and T_2_ triangles in Fig. 4(b) are plotted in Fig. 4(c) at the normalized frequency 0.361 and 0.339*c*/*a* with the same wave vector ${k}_{x}=-0.3(2\pi /a)$, respectively. The modes are confined at the corresponding interface. Moreover, a zoom-in look at the highlighted regions (black dashed lines) reveals more details of the vortex nature of valley states at T_1_ and T_2_ points. The distributions of the calculated time-averaged Poynting vectors are shown in Fig. 4(d), where the positive and negative orbital angular momentum can be found to be carried by ${{\rm{\Phi }}}_{{\rm{I}}\,\,{\rm{II}}}^{\uparrow }$ and ${{\rm{\Phi }}}_{{\rm{I}}\,{\rm{I}}\,{\rm{I}}}^{\downarrow }$ states. The color of interface state dispersions reflects the sign of orbital angular momentum carried, where the states with positive and negative orbital angular momentum are in purple and green respectively.

**Figure 5:**
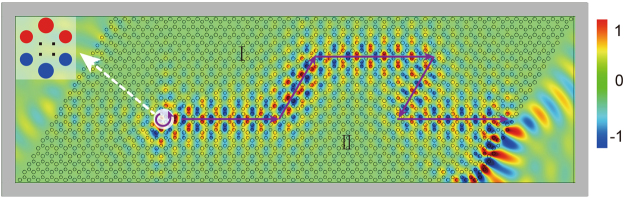
The simulated *E*_*z*_ field distribution of topological interface state at normalized frequency 0.349*c*/*a*. The interface state propagates forward unidirectionally along the channel with weak backscattering excited by source with optical vortex index *m* = 1. The insert is the zoom-in position of the excitation source.

From Fig. 4, one can easily recognize that the mode allowed on the interface between PC I and II depends on the interface structure. Moreover, the propagating direction of the interface state also depends on the orbital angular momentum carried. The upward propagation Φ_I II_ mode is ${{\rm{\Phi }}}_{{\rm{I}}\,{\rm{II}}}^{\uparrow }$ which carries the positive orbital angular momentum and thus can be effectively excited by a source with m = 1 optical vortex index. Similar sources were used to experimentally excite topological valley interface states^30,37^. We perform the finite-element simulations to visualize the topological interface states. As depicted in Fig. 5, we use a PC structure sized of 21 × 51 unit cells to construct a domain wall. PC II is located below PC I, which both have identical parameters as in Fig. 1. As one wish to excite the mode to propagate towards right, ${{\rm{\Phi }}}_{{\rm{I}}\,{\rm{II}}}^{\uparrow }$ shall be excited. We apply perfectly-matched-layer (PML) boundary conditions (in gray) around the domain wall to avoid reflection from the boundaries. The source with optical vortex index m = 1 (indicated in white) at the frequency 0.349*c/a* is launched at the center of interface between PC I and II, where a zoom-in picture of its position and neighboring PC structures is inserted in Fig. 5. The simulated *E*_*z*_ field is concentrated along the domain wall and exponentially decays into the bulk on both sides. The topological interface state propagates unidirectionally and travels tens of wavelength without noticeable attenuation. Backscattering is nearly prohibited in despite of four sharp turns on the loop. Other than a refracted beam to free space at the exit port in the domain wall, the exit beam also excites the interface state along the zigzag interface between the PC II and air^39^. If a stimulus with optical vortex index *m* = −1 is considered, the interface state will transmit backward. Because the excitation is not the eigen mode of the interface state, some electromagnetic energy leaks to the left side. Nearly unidirectional performance can be found in nearly the whole frequency range of the interface state dispersion.

### Bulk Valley Devices

**Figure 6:**
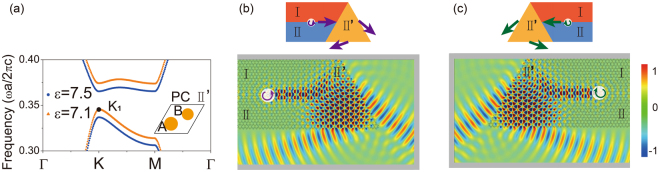
(**a**) The band dispersion with completed gap for PC structure II′. The frequency of *K*_1_ valley is slightly higher than that of PC II because of its lower relative permittivity $\varepsilon =7.1$. The schematic diagrams of the bulk valley filter are shown in (**b**) and (**c**) under top view. (**b**,**c**) The *E*_*z*_ electric fields show that the valley polarization for *K*_1_/${K^{\prime} }_{1}$ point of PC II′ is excited by the topological interface state i.e. pseudospin up/down state at the frequency 0.345*c*/*a*, respectively.

In valleytronics research, scientists are targeting to design devices based on the valleys degrees. It is more than merely a channel to conduct valley polarized information. Therefore, it will be important to selectively excite bulk valley states, aka valley filters, and couple them into/out of the device, which so far is still challenging in the electronic system^3,16^. Here, firstly a bulk valley filter is realized based on PC scheme. We construct a new PC II′ as shown in the inset panel of Fig. 6(a), whose dielectric parameters are relative permittivity $\varepsilon =7.1$, the cylinder diameters ${d}_{A}=0.4a$ and ${d}_{B}=0.333a$. The only difference between PC II′ and II structures is that the former uses materials with a smaller permittivity. In the band structure of PC II′ (see Fig. 6(a)), the normalized frequency of *K*_1_ (black solid circle) is 0.345*c*/*a*, which is slightly higher than that in PC II (0.337*c/a* in Fig. 1(e)). We design a geometry shown in the top panel of Fig. 6(b) to filter one of the two bulk valley states. A domain wall is constructed by PC I (red region) and II (blue region) to conduct information of valley and it is connected to PC II′ of triangular geometry (orange region). In Fig. 6(b), a topological interface state with pseudospin up ${{\rm{\Phi }}}_{{\rm{I}}\,{\rm{II}}}^{\uparrow }$ excited by the chiral source (*m* = 1) at the frequency 0.342*c*/*a*, which is around the *K*_1_/${K^{\prime} }_{1}$ point of PC II′. From the discussion in the previous session, this excited interface state propagates forwardly to impinge on PC II′. Because the mode profile of the bulk *K*_1_ state is with optical vortex index *m* = 1, the topological interface state ${{\rm{\Phi }}}_{{\rm{I}}\,{\rm{II}}}^{\uparrow }$ couples efficiently with the *K*_1_ valley state of PC II′. We confirm that the *K*_1_ bulk valley state is well excited by examining the internal phase distribution and analyzing the refracted beam direction out of PC II′ triangular structure. The unidirectional interface state ${{\rm{\Phi }}}_{{\rm{I}}\,{\rm{II}}}^{\uparrow }$ valves the bulk valley selection. During this process, the ${K^{\prime} }_{1}$ valley of PC II′ is suppressed because it has an optical vortex index *m* = −1 and its mode profile can only be stimulated by light incidence of ${{\rm{\Phi }}}_{{\rm{I}}\,{\rm{II}}}^{\downarrow }$ with negative orbital angular momentum. If a connecting domain wall is changed accordingly to accommodate ${{\rm{\Phi }}}_{{\rm{I}}\,{\rm{II}}}^{\downarrow }$ state stimulated by an *m* = −1 source, the ${K^{\prime} }_{1}$ valley-polarization (distinguished from the direction of refraction beams) is excited as shown in Fig. 6(c). We have to emphasize that the introduction of PC II′ structure is solely to ease the demonstration of valley filter effect. If the working frequency is tuned to excite *K*_2_/${K^{\prime} }_{2}$ state, similar valley filtering can be realized. Therefore, our designed devices serve well as valley filters. One can excite a specific bulk valley state by either exciting a specific chiral source or tuning the working frequency. It might be used as information transmission by valley PC. We must emphasize that the using of the chiral source to excite topological interface is for a better demonstration the selection of valley states. Even if an arbitrary source is located at one end of the domain wall between PC I and II, as the orbital angular momentum carried of interface state is picked up by its propagating direction, the bulk valley selection can still be realized^29,40^.

**Figure 7:**
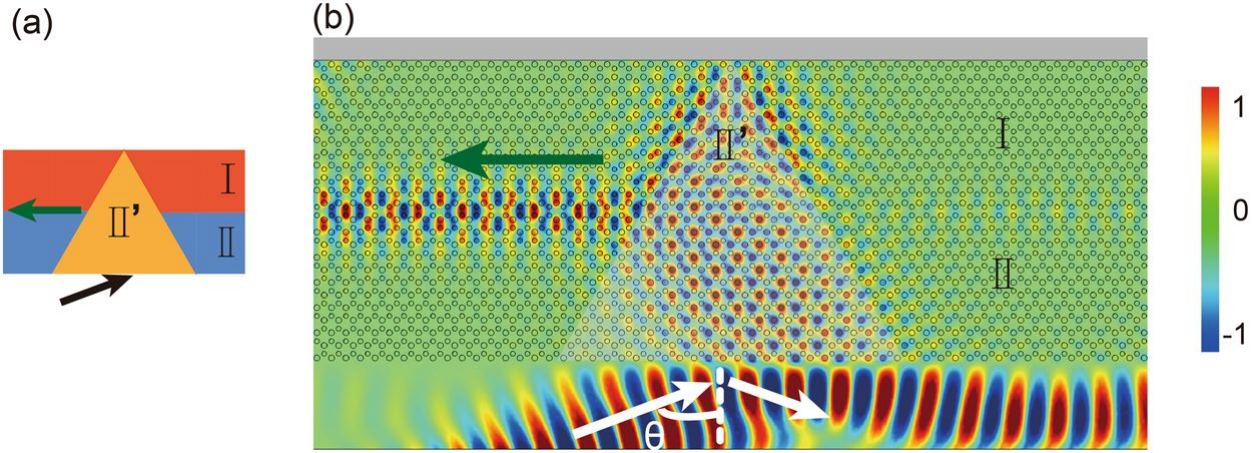
(**a**) The schematic of valley coupler. (**b**) The ${K^{\prime} }_{1}$ valley state, excited selectively by incident Gaussian beam, couples to the interfacial domain wall of PC system and the pseudospin down ${{\rm{\Phi }}}_{{\rm{I}}\,{\rm{II}}}^{\downarrow }$ state propagates along the left channel.

Valley polarized transmission and filtering can be excited in multiple methods. We here realize a valley coupler by exciting bulk valley state through external light incidence and selectively coupling to domain walls for valley polarized transport. The schematic of the valley coupler is shown in Fig. 7(a). The ${K^{\prime} }_{1}$ valley state of PC II′ is excited by the incident Gaussian beam displayed in Fig. 7(b). Because the incident angle *θ* matches with momentum conservation and the selected frequency is at the lower branch, only the ${K^{\prime} }_{1}$ state with *m* = −1 optical vortex index is excited. This state matches with the allowed left-handed propagating ${{\rm{\Phi }}}_{{\rm{I}}\,{\rm{II}}}^{\downarrow }$ pseudospin state. Thus, the topological interface state emerges and propagates along this interface channel. The transmittance to the right domain wall is prohibited, because it only can accommodate forward propagating ${{\rm{\Phi }}}_{{\rm{I}}\,{\rm{II}}}^{\uparrow }$ state. Obviously, if the beam’s incident angle is reversed, the direction of the valley coupler can be reversed.

## Conclusions

In conclusion, we have studied the exotic valley properties of PCs. Valley polarization related photonic devices are demonstrated. By engineering the inversion symmetry of a honeycomb lattice PC, a direct analogy to AB/BA stacking bilayer graphene is found. Relation of chirality to inequivalent valley states is investigated and is related to optical vortex indices. The valley states are selectively excited by using the external stimuli, i.e. the chiral source with different optical vortex indices or incident Gaussian beams at special angles. Topological unidirectional interface states are demonstrated to exist at the domain wall of two inverted PCs with none zero valley Chern numbers. Due to the similar optical vortex index carried by both the interface states and bulk valley states, coupling between them is possible. Consequently, valley devices including bulk valley filter and valley coupler are achieved by manipulating the geometry between interfacial domain walls and bulk valley materials. Our results not only suggest a promising platform of future application of valley PCs, but also help the design of optical or telecommunication devices using valley degree of freedoms.

## Methods

Throughout this paper, all numerical simulations are performed by COMSOL Multiphysics, 4.3a, RF module, commercial software based on finite element method (www.comsol.com).

### Data availability

The data that support the findings of this study in the paper are available from the corresponding author upon reasonable request.
